# Validation of a risk prediction model for COVID-19: the PERIL prospective cohort study

**DOI:** 10.2217/fvl-2023-0036

**Published:** 2023-11-07

**Authors:** Shahd A Mohammedain, Saif Badran, AbdelNaser Y Elzouki, Halla Salim, Ayesha Chalaby, MYA Siddiqui, Yehia Y Hussein, Hanan Abdul Rahim, Lukman Thalib, Mohammed Fasihul Alam, Daoud Al-Badriyeh, Sumaya Al-Maadeed, Suhail AR Doi

**Affiliations:** ^1^Department of Population Medicine, College of Medicine, QU Health, Qatar University, Doha, Qatar; ^2^Department of Plastic Surgery, Hamad Medical Corporation, Doha, Qatar; ^3^Department of Internal Medicine Hamad General Hospital Hamad Medical Corporation, Doha, Qatar; ^4^Department of Public Health, College of Health Sciences, QU Health, Qatar University, Doha, Qatar; ^5^Department of Biostatistics, Faculty of Medicine, Istanbul Aydin University, Istanbul, Turkey; ^6^College of Pharmacy, QU Health, Qatar University, Doha, Qatar; ^7^Department of Computer Science, College of Engineering, Qatar University, Doha, Qatar

**Keywords:** COVID-19, disease severity, prognosis, risk prediction

## Abstract

**Aim:** This study aims to perform an external validation of a recently developed prognostic model for early prediction of the risk of progression to severe COVID-19. **Patients & methods/materials:** Patients were recruited at their initial diagnosis at two facilities within Hamad Medical Corporation in Qatar. 356 adults were included for analysis. Predictors for progression of COVID-19 were all measured at disease onset and first contact with the health system. **Results:** The C statistic was 83% (95% CI: 78%–87%) and the calibration plot showed that the model was well-calibrated. **Conclusion:** The published prognostic model for the progression of COVID-19 infection showed satisfactory discrimination and calibration and the model is easy to apply in clinical practice.d

In December 2019, the novel coronavirus SARS-CoV-2, responsible for COVID-19, emerged in Wuhan city, China. It has since rapidly spread all over the world. By 19 January 2020, four countries had reported laboratory-confirmed cases of COVID-19. Four days later on 23 January 2020, the city of origin was placed under lockdown, and multiple countries soon followed suit [1]. The WHO announced COVID-19 as a pandemic in March 2020 and subsequently over 110 million confirmed cases were recorded, with more than 2.45 million deaths [2]. The pandemic has had a massive negative impact on the global economy and incurred enormous detrimental effects on mental health worldwide [3,4]. The pandemic had also placed a catastrophic burden on healthcare systems and a reliable validated tool to stratify patients according to the risk of progression to severe illness from disease onset was urgently needed to guide resource allocation.

Since the start of the pandemic, various prediction models have been created using patients' demographics, medical history, signs and symptoms or laboratory investigations to predict COVID-19 prognosis. These models can be categorized into two main types: those that were developed from variables measured at disease onset and those that used variables measured during the course of the illness [5–8]. According to a systematic review by Wynants *et al.*, most of the existing prediction models for COVID-19 lacked validation, were inadequately reported, or were at high risk of bias, which has discouraged their use because of the limitations associated with the reliability of predictions from such models [9]. We had also developed one such tool [10], and in this paper we aim to generate evidence of its reliability as a tool to triage COVID-19 patients at disease onset and thus provide a useful tool to alleviate the burden on the healthcare system.

Many attempts have been made to stratify the risk of severe COVID-19 at disease onset with subsequent severity defined as the need for intensive care unit (ICU) admission, need for invasive ventilation or death [11]. The latter definitions of progression have clear advantages because they use hard end points that are reliably measurable. Biomarkers are an obvious choice for such prediction models but there has not been such a model that has been extensively validated. The advantage of using biomarkers for patient triage is that it is likely to be more objective and reliable than patient-reported symptoms. In this study, we aim to externally validate our previously developed and biomarker based Kuwait Prognosis Indicator (KPI) model [10].

## Patients & methods

### Study population of the external validation cohort

**Figure 1.**
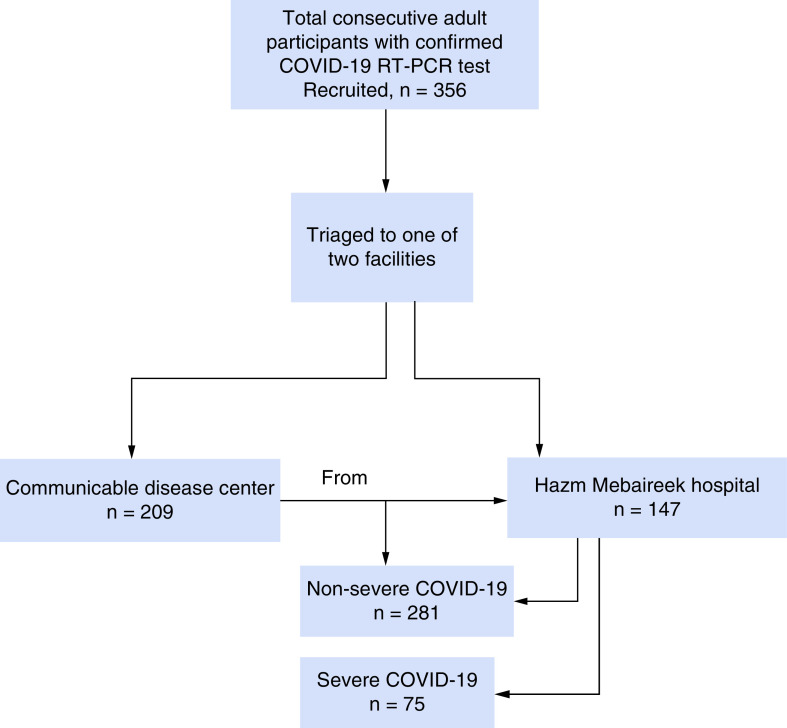
Flow of patient recruitment.

A prospective cohort study, Predicting Risk Early in COVID-19 (PERIL), was undertaken to validate the KPI model. Between 23 August 2020 and 22 November 2020, there were 356 adult patients recruited at their initial diagnosis at two facilities within Hamad Medical Corporation in Qatar: the Communicable Disease Center (CDC), which dealt with referral cases that are asymptomatic at diagnosis and then allocated patients to an isolation facility, and the Hazm Mebaireek General Hospital, a secondary care facility that receives patients considered to be symptomatic at presentation for observation. In both centers, biomarkers were measured on the day of referral (either for symptom onset or positivity on contact tracing) to these centers (see recruitment flow chart in Figure 1). Qatar CDC guidelines were followed for all the recruited patients, whether they were isolated at home or within special facilities, or they were admitted to the hospital for observation.

### Ethical consideration

This study was approved by the medical ethics committee of Qatar University and Hamad Medical Corporation (protocol nos. QU-IEB 1434-E/20 and MRC 05-137, respectively) and written informed consent was obtained from all participants. Results have been reported to conform with the transparent reporting of a multivariable prediction model for individual prognosis or diagnosis (TRIPOD) statement [12].

### Predictor assessment

Predictors for the progression of COVID-19 (age, serum procalcitonin, CRP, lymphocyte percentage, monocyte percentage and serum albumin) were all measured at disease onset and first contact with the health system. These predictors were selected after a careful review of the COVID-19 literature as the most relevant for a risk prediction model with a focus on biomarkers. Participants were identified either through contact tracing, routine testing or self-reported symptoms. Table 1 provides information on the predictors and cutoffs used.

**Table 1.** The KPI prediction model.

Kuwait prognosis Indicator score for COVID-19. Please give your patient zero points if criterion not met	
Criterion	Points
Age ≥ 41 years	4
CRPs ≥ 7 mg/l	2
Procalcitonin ≥ 0.05 ng/ml	16
Lymphocyte percent ≥ 31.5%	-9
Monocyte percent ≥ 9.2%	-8
Albumin ≥ 39.5 g/l	-15
Total	

Low progression risk total ≤ -7.

Uncertain progression risk total -6 to 15.

High progression risk total ≥16.

### Outcome assessment

Severe COVID-19 was defined as progression to ICU admission, need for invasive ventilation or in-hospital death. This was considered the most pragmatic and reliable definition of severity [11].

### Sample size

For studies validating prognostic models, there is no solid sample size recommendation, but a minimum of 100 patients with events and at least 100 patients without events has been suggested [13].

### Missing data

In the event of missing data, the use of multiple imputation by chained equations (MICE) was planned. This method imputes missing data multiple times to account for uncertainty.

### Statistical analysis

Baseline characteristics of patients in the PERIL cohort were reported as median and interquartile range or number and percent and were stratified by severity status. The original prognostic model was applied exactly to our study cohort as it was published previously [10]. Discrimination was assessed using Harrell's C statistic. This is equal to the area under the receiver operating characteristic curve, and it indicates that when the model predicts that a participant will have a high risk for severe disease, the participant is more likely to develop severe disease.

Calibration of the validated model was assessed by comparing the calculated and predicted probabilities of severity for each individual with the actual observed outcomes in the calibration plots. If the calculated predicted probabilities equal to the actual proportions for the groups, then the model is well-calibrated. Statistically, the well-calibrated model will have a calibration plot with an intercept of 0 and a slope of 1, and the groups of predicted probabilities should lie relatively close to this line. All analyses were performed using Stata MP 15.1 (StataCorp, TX, USA).

### Comparison of development & validation studies

The setting, eligibility criteria and predictors in this validation study were similar to those in the development study [10]. However, the outcome definition we used in the development study was a composite outcome that combined soft and hard end points. In this validation study, we only used hard end points and no soft end points were collected.

## Results

### PERIL cohort

Three hundred-fifty-six participants were recruited (Figure 1). Of these participants, 209 were recruited through the CDC (59%) and 147 through the Hazm Mebaireek General Hospital (41%). After a complete chart review and confirmation of the data collected, no missing data was encountered. Table 2 shows the baseline characteristics of the participants. Severe COVID-19 was diagnosed in 75 (21%) participants, of whom 7 (9%) died. Compared with the development study [10], the distribution of age (median 39 years vs 45 years) and sex of participants (males 72.5 vs 75%) were also similar, as were the percentage who died (2.9 vs 2%).

**Table 2.** Baseline characteristics.

Characteristic	All	Percent/IQR	Non-severe (n = 281)	Percent/IQR	Severe (n = 75)	Percent/IQR
Demographics						
Age (years)	45	36.5 to 56	34	44 to 55	49	43 to 58
Male	267	75%	193	68.7%	74	98.7%

Comorbidities						
Cardiovascular disease	124	34.8%	90	32.0%	34	45.3%
Diabetes	112	31.5%	75	26.7%	37	49.3%
COPD/Asthma	11	3.1%	8	2.8%	3	4.0%
Renal disease	18	5.1%	8	2.8%	10	13.3%

Biomarkers						
Lymphocyte %	23.2	14 to 33.8	28	17.9 to 37.15	11.7	7.2 to 18.5
Monocyte %	7.7	5.3 to 10.9	8.4	6.05 to 11.4	4.8	3.2 to 8.2
CRP mg/l	18.1	3.6 to 106.2	8.65	2.75 to 60.95	150.3	69.2 to 229.4
Albumin g/l	37	32 to 40	34	38 to 41	28	32 to 34
Procalcitonin (ng/ml)	0.07	0.04 to 0.21	0.06	0.03 to 0.11	0.65	0.18 to 1.28

Medical course						
Significant medications	185	52%	110	39.1%	75	100.0%
X-ray changes	208	58.4%	135	48.0%	73	97.3%
Length of stay (days)	6	0–14	0 0 to 10		14 20 to 28	
ICU admission	75	21.1%				
Death	7	2.0%				

Prognostic score						
KPI score	6	-10 to 22	-1	-13 to 14	22	18 to 22

Data are shown as median and interquartile range or number and percent.

COPD: Chronic obstructive pulmonary disease; ICU: Intensive care unit; IQR: Inter-quartile range; KPI: Kuwait Prognosis Indicator.

### Calibration & discrimination of the prognostic model

**Figure 2.**
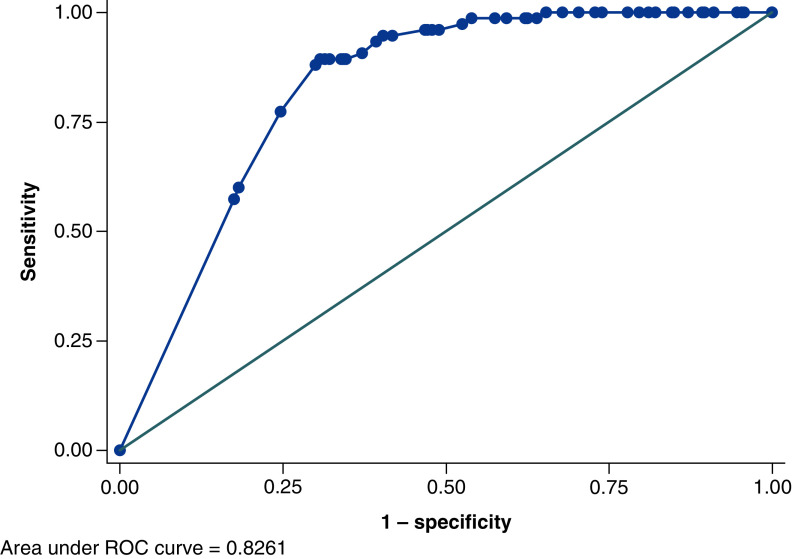
Receiver operating characteristics plot depicting discrimination of the Kuwait Prognosis Indicator score. ROC: Receiver operating characteristic.

**Figure 3.**
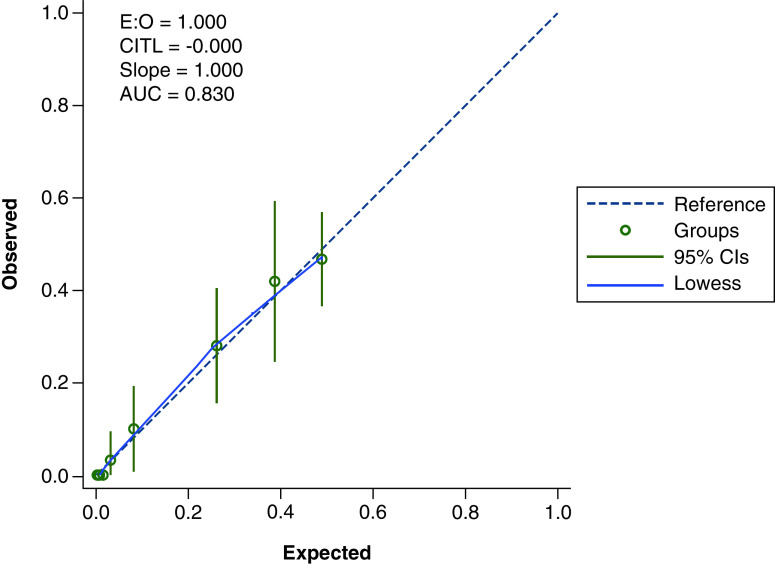
Plot depicting Kuwait Prognosis Indicator model calibration. AUC: Area under the curve; CI: Confidence interval.

The C statistic for the model was 0.83 (95% CI 0.78–0.87; Figure 2). The model showed good calibration, as evidenced both by the calibration plot and the calibration slope and intercept (Figure 3). It was not possible to split the predicted probabilities into ten groups so the calibration plot had fewer than 10 points. The model included only a few categorical variables (i.e. was a sum score model) in which a limited number of predicted probabilities were possible [14].

Clinical performance was evaluated using interval likelihood ratios (Table 3). In the low-risk group, the likelihood ratio for progression was 0.036 (95% CI: 0.005–0.251). In the high-risk group, the likelihood ratio for progression was 3.138 (95% CI: 2.472–3.984).

**Table 3.** Interval likelihood ratios for the KPI model.

Interval of KPI score	Severe COVID	Not severe COVID	Likelihood ratio	95% CI
-32 to -7 (Low risk)	1	105	0.0356	0.00504 to 0.251
-6 to 15 (Intermediate risk)	16	106	0.564	0.356 to 0.892
16 to 22 (High risk)	58	69	3.138	2.472 to 3.984
Total	75	280		

CI: Confidence interval; KPI: Kuwait Prognosis Indicator.

## Discussion

In the development study [10], a KPI model was created to predict progression to severe disease, thus enabling pre-stratification of patients with COVID-19 according to their risks of progression. The KPI model was based on age and five laboratory tests at presentation. The development study assessed the model to have an area under the curve (AUC) of 0.83 (95% CI: 0.78–0.89) and the model was externally validated on an open-source dataset demonstrating an AUC of 0.89 (95% CI: 0.85–0.92). In this prospective validation study, we aimed to prospectively validate the KPI model for early prediction of the risk of progression to severe COVID-19 as measured by a composite of three hard outcomes – ICU admission, need for invasive ventilation, or death. Despite this validation being in a different health system, the C-statistic in our model showed similar discrimination as in the development cohort with an AUC of 0.83 (95% CI: 0.78–0.87). The model also showed good calibration results.

Many prediction models have been created since the pandemic started [15–39]. Several of these have used variables that were not useful for risk stratification [5–8]. Of those that considered baseline patient variables, some reported less solid outcomes such as hospitalization, a progression of signs and symptoms, or imaging results (Table 4) making these less reliable and not as useful to decision-making.

**Table 4.** Prediction models that have mortality or ICU admission as an outcome.

Variables included	AUC	External validation AUC	Calibration	Ref.
Demographics Biomarkers Vital signs	0.80 to 0.84	0.72 to 0.83	Yes	[28]
Demographics History Biomarkers Vital signs	0.74 (ICU) 0.83 (mortality)	–	–	[29]
Demographics History	0.80	–	–	[37]
Demographics History	0.83	–	–	[38]
Demographics Vital signs Biomarkers	0.83 to 0.86	0.83 to 0.85	Yes	[34]
Demographics History Biomarkers Vital signs	0.79	0.77	Yes	[30]
Demographics Vital signs Biomarkers	0.798	–	–	[31]
Demographics History Signs and symptoms	0.897	0.885	–	[35]
Demographics Biomarkers	0.90	0.84 to 0.93	Yes	[32]
Demographics History Biomarkers Vital signs	0.87	–	Yes	[39]
Biomarkers	0.92	–	–	[33]
Demographics Biomarkers	0.83	0.89 0.83	Yes	[10]

These studies have been excluded from the table as they reported soft outcomes [15–27].

AUC: Area under the curve; ICU: Intensive care unit.

In keeping with our model, eleven prediction models (Table 4) measured patient variables at baseline and used hard outcomes similar to ours and many of these used one or more of the biomarkers used in the KPI model [28–33]. Vaid *et al.* created an in-hospital mortality and critical events prediction model for COVID-19 using demographics, biomarkers and vital signs, enrolling 1514 patients and reporting that higher age and CRP were among the strongest predictors of mortality [28]. Another prediction model developed by Zhao *et al.* used data from 454 patients and reported procalcitonin to be an important predictor of both ICU admission and death [29]. Moreover, the bootstrap analysis in the model created by Altschul *et al.* revealed that from the mortality predictors, age, oxygen saturation, urea nitrogen, CRP, international normalized ratio and procalcitonin were reproducibly selected in more than 70% [31]. However, procalcitonin was later removed from the model due to a large number of missing values. In another model developed by Wu *et al.* in Wuhan, the variables that were selected in the model included age, lymphocyte (proportion) and CRP, which are also found in our KPI model [32]. Hu *et al.* identified albumin at admission to be an important early predictor of severe COVID-19 and constructed a prediction model that used only two biomarkers – albumin and lymphocyte count [33]. The range of discrimination in all these models in terms of the AUC was between 0.74 and 0.92 and five of these were externally validated [28,30,32,34,35]. Among these models, the Wu *et al.* model and the KPI model are the only ones that have now been validated in two different countries [10,32].

Wu *et al.* developed the model using demographic and laboratory variables and reported an AUC of 0.86 in the training dataset (n = 239) and an AUC of 0.9 in the testing dataset (n = 60) [32]. The model was further externally validated on five test datasets, which showed AUCs ranging from 0.84 to 0.93 with accuracies ranging from 74.4 to 87.5% in China, Italy, and Belgium. Although the model shows better discrimination than the KPI model, it has a higher risk of bias due to the smaller number of events per variable (EPV <10) [36].

A limitation of our model is the use of categorical data that may have resulted in decreased predictive ability, but this was a pragmatic choice. Nevertheless, the discrimination is comparable to what has been previously reported. In addition, the use of biomarker assays may be a limitation in rural and remote communities where they may not be available. A strength of this validation study is that patients were prospectively followed, and all tests and outcome measurements were conducted prospectively.

## Conclusion

In conclusion, we externally validated and calibrated our KPI model, presenting an easy-to-use six variable interface that can aid clinicians in accurately stratifying patients likely to progress to more severe disease such as ICU admission, need for invasive ventilation and death. The model used measures collected early at diagnosis, and given that risk is a continuum, this model may also be expected to define moderate-severity cases that need supportive care (e.g., oxygen), but do not end up in the ICU. The performance of the KPI model within the validation cohort showed that this tool could speed up patient triage and will therefore optimize the allocation of resources in health systems overburdened by a COVID-19 wave.

**Summary points**

- The Kuwait Prognosis Indicator (KPI) is a novel biomarker-based risk prediction tool for COVID-19.
- The KPI tool contains hard biomarker predictors as opposed to soft predictors such as patient signs and symptoms.
- The KPI is easy to use and is now validated.
- The KPI has good discrimination of those that will need ICU care.
